# The association of rs187238, rs19465518 and rs1946519 IL-8 polymorphisms with acute kidney injury in preterm infants

**DOI:** 10.37796/2211-8039.1231

**Published:** 2021-12-01

**Authors:** Fiva Aprilia Kadi, Tetty Yuniati, Yunia Sribudiani, Dedi Rachmadi

**Affiliations:** a Department of Child Health, Faculty of Medicine, Universitas Padjadjaran, Dr Hasan Sadikin General Hospital, Bandung, Jalan Pasteur no. 38 Bandung 40161, West Java, Indonesia; b Department Biomedical Science, Biotechnology, Molecular Biology and Genetic Faculty of Medicine, Universitas Padjadjaran, Bandung, Jalan Eiykman no. 38 Bandung 40161, West Java, Indonesia

**Keywords:** Acute renal injury, Polymorphism, Premature, Serum IL-18, Urine IL-18

## Abstract

**Background:**

Interleukin 18 (IL-18) promoter polymorphisms (−656G > T, −607C > A, and −137G > C) affect serum IL- 18 (sIL-18) levels and are associated with renal injury.

**Purpose:**

This study aimed to determine the diagnostic utility of sIL-18 and urine IL-18 (uIL-18) as biomarkers for acute kidney injury (AKI) and analyse the association of *IL-18* polymorphisms to AKI in preterm infants.

**Methods:**

Blood and urine samples were collected from 56 preterm infants with AKI and 56 without AKI to measure serum creatinine (SCr), sIL-18, and uIL-18. Genotyping of polymorphisms was performed and analysed, with AUC-ROCs analysis used to evaluate the diagnostic utility of s-/uIL-18 levels.

**Results:**

The median sIL-18 and uIL-18 levels were significantly higher than those without AKI. For a cutoff of >132 pg/mL, the sIL-18 expression had sensitivity and specificity of 80.36% and 60.71%, respectively, while for uIL-18, a cutoff of >900.7 pg/mL had sensitivity and specificity of 51.79% and 78.57%, respectively. The odds ratio of sIL-18 and uIL-18 to predict AKI in preterm infants was 5.89 (95%CI:2.31–15.02) and 4.15 (95%CI:1.58–10.89), respectively. The polymorphisms −137G > C and −656G > T were significantly associated with sIL-18 expression.

**Conclusion:**

Serum and urine IL-18 levels are risk factors for and a moderate predictor of AKI in preterm infants.

## 1. Introduction

According to CDC data in 2019, the incidence of preterm birth is 10–15%, with a high rate of morbidity, especially at a gestational age of less than 33–34 weeks due to the immaturity of the organs [1–6]. Several studies have shown that acute kidney injury (AKI) in preterm neonates has short-term and long-term effects on the kidney [1–9]. However, there is no consensus on the best practice for the diagnosis of neonatal AKI, partially due to neonatal serum creatinine (SCr) levels in the first 48–72 h of life being influenced by the maternal creatinine level [8–12]. The latest diagnostic criteria for AKI as proposed by the Acute Kidney Injury Working Group of KDIGO (Kidney Disease: Improving Global Outcomes) is based in absolute increase of sCr, at least 0.3 mg/dL (26.5 μmol/L) within 48 h or by a 50% increase in sCr from baseline within 7 days, or a urine volume of less than 0.5 mL/kg/h for at least 6 h [12, 13].

Preterm birth is associated with maternal inflammation during pregnancy, marked by an imbalance of interleukins (IL), particularly, IL-6, IL-10, IL-18 and tumour necrosis factor-alpha (TNFα) [14–16]. It has been reported that IL-18 is associated with AKI, with adults having an increase in IL-18 after 24 h of renal ischemia or decreased perfusion, especially in the proximal tubule [14–17]. A previous study in neonatal term infant showed that urine IL-18 (uIL-18) can be used as a predictor of AKI with sensitivity more than 90% [18], another study in neonatal critically ill had a sensitivity of 64% and a specificity of 92% [19]. Furthermore, IL promoter polymorphisms are known to affect cytokine expression levels. Five single nucleotide polymorphisms (SNPs) in the promoter area and exon 1 of IL-18 gene have been identified, i.e. −137 G > C, −607C > A, −656 G > T, +113 T > G and +127C > T, with −137 G > C (rs187238), −607C > A (rs1946518), and −656 G > T (rs1946519) affecting IL-18 expression [20–28].

## 2. Purposes

This study aims to determine the utility of sIL-18 and urine interleukin 18 (uIL-18) as biomarkers of AKI in preterm infants and whether the −137G> C (rs187238), −607C > A (rs1946518) and −656 G > T (rs1946519) polymorphisms are associated with the expression levels of s-/uIL-18 and AKI phenotype in preterm infants.

## 3. Methods

### 3.1. Study design and subjects

This case–control study recruited 112 neonates born at 30–36 weeks of gestation, with written informed consent from parents obtained prior to inclusion. Sample size was determined based on formula for comparing two proportion with significance level 5% (one tailed); power of test 80%; and P_1_ = proportion of genotype IL-18 allele C = 0.19 [20] (P1 is the highest Minor Allele Frequency among three polymorphism of IL-18 promoter polymorphism), with OR = 3; and for formula P_1_ = P2 * OR/((1+P_2_ (OR-1)) = 0,57/(1.38) = 0.42. From the formula sample size derived at least n = 47 for each group. By setting a drop out = 10%, the minimum sample size will be: 1/(1–0,10) * 47 = 53 per group.

The study protocol was approved by the Faculty of Medicine, University of Padjadjaran Bandung (Ethical clearance number 72/UN6.KEP/EC/2019). The subjects were divided into two groups: the case group (56 infants with AKI) and the control group (56 infants without AKI) and the diagnosis of AKI was according to the neonatal-KDIGO (nKDIGO) criteria, that is, an increase in serum creatinine of −0.3 mg/dL in the last 48 h or a 150–200% increase in serum creatinine level compared to baseline, and a urine volume of less than 0.5 cc/kg/h. Samples were collected consecutively. The exclusion criteria were oligohydramnion, maternal infection, and multiple congenital abnormalities. Trained health personnel were collected blood samples (1 mL) from the umbilical vein and urine samples were obtained via a urinary catheter. All biological samples were directly transported to the clinical laboratory of Dr Hasan Sadikin Hospital for biochemical analysis and the molecular genetics laboratory for DNA isolation within 2 h after collection.

### 3.2. Measurement of serum creatinine and urine/serum IL-18

Serum creatinine was measured within the first 24 h and repeated on day 3 (72–96 h post-natal) using a modified kinetic technique CRE2 method (Dimension Vista System Creatinine/Siemens Healthcare Diagnostic)^R^. IL-18 levels were measured in the serum and urine of the subjects in the first 24 h using an ELISA.

### 3.3. Genotyping of IL-18 promoter polymorphisms

DNA was isolated from 300 to 500 μl of blood using a Quick-DNA Miniprep Plus kit (Zymo Research, CA, USA). Polymerase chain reaction (PCR) was performed using 50–100 ng of DNA, 25 μl of MyTaq™ HS Red Mix (Bioline), 1 μl of 10 pmol/μl of each forward and reverse primers and MQ water (Millipore; to total volume of 50 μl). Primers used to amplify each SNPs in this experiment are presented in Table S1 and the PCR was performed on a touch-down PCR machine with an annealing temperature ranging from 65°C to 55°C for a total of 35 cycles (Table S2). The PCR products were subjected to gel electrophoresis (1.5% agarose gel) and purified for Sanger sequencing on an ABI-3500. The sequencing results were analysed using Bioedit Software.

### 3.4. Statistical analysis

Descriptive analyses were performed with data expressed as frequency and percentage for categorical data. Statistical analysis was performed using an independent t-test or the Mann–Whitney U test. Univariate statistical analysis continued with bivariate logistic regression was conducted to analyse prominent risk factors that relate simultaneously to the outcome. Area under the receiver-operating characteristic curve (ROC-AUC) analysis, positive predictive value (PPV) and negative predictive value (NPV) were used to evaluate the diagnostic utility of the serum and urine IL-18 level. The risk was analysed using the odds ratio (OR) with a 95% confidence interval and a two-tailed p < 0.05 was considered significant.

## 4. Results

### 4.1. Subject characteristics

There were no significant differences in gender, gestational age and birth weight in preterm infants with and without AKI. The level of serum IL-18 and urine were significantly higher in preterm infants with AKI than non-AKI infants (p < 0.05) (Table 1).

### 4.2. Expression of sIL-18 and uIL-18 in preterm infants

**Fig. 1:**
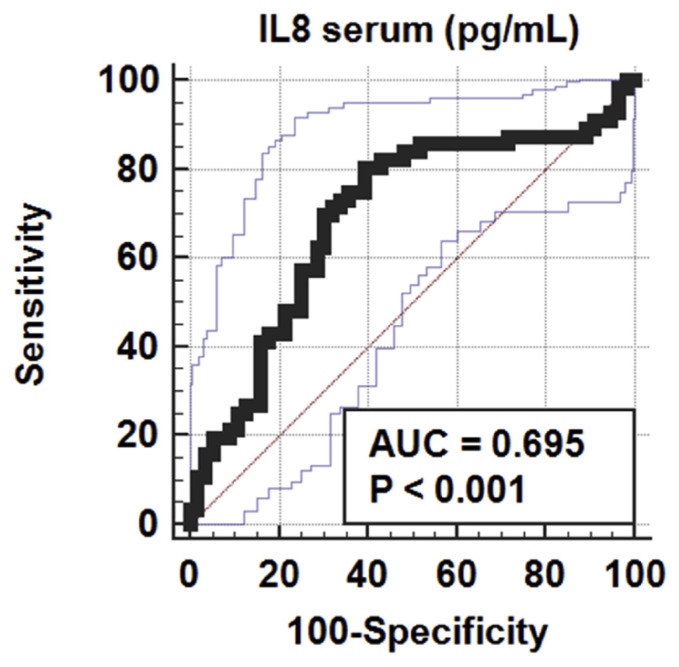
ROC curve for the utility of serum IL-18 for the prediction of AKI in preterm infants. AUC = 0.72 (95%CI: 0.494–0.863) with a cut-off point >132 pg/mL***:*** sensitivity 80.36%; specificity 60.71%.

**Fig. 2:**
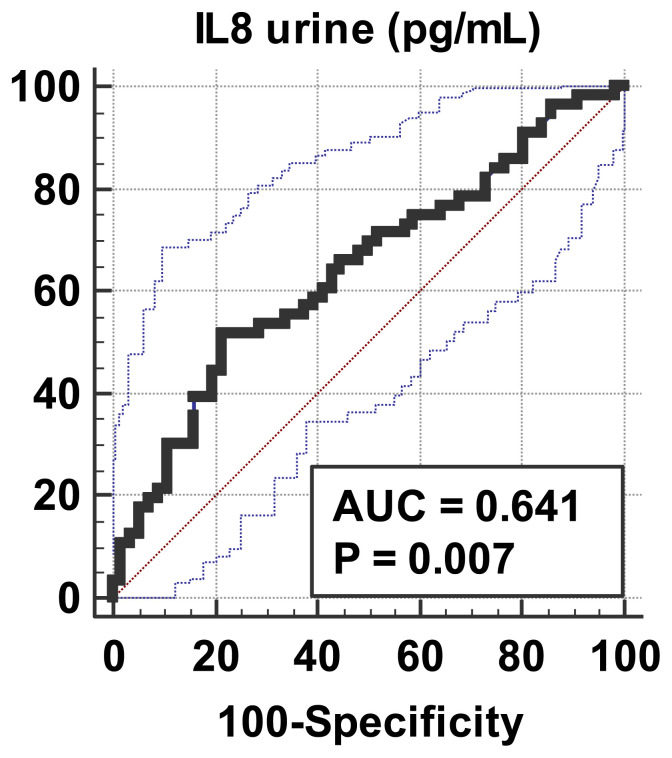
ROC curve of urine IL-18 used as a predictor of AKI in preterm infants. AUC = 0.62 (95%CI: 0.532–0.718) with a cut-off point >900.7 pg/mL: sensitivity 51.79%; specificity 78.57%.

Based on the differences in sIL-18 and uIL-18 levels in preterm infants with and without AKI, a cut off point of IL-18 to predict AKI was determined by AUC-ROC analysis (see Figs. 1, 2). The results showed that the AUC-ROC for the utility of sIL-18 for AKI in preterm infants was 0.72 (95%CI: 0.494–0.863). The cut off point was >132 pg/mL, with a sensitivity of 80.36% and specificity of 60.71%. This test had a PPV of 58.44% and NPV of 75.56%. For uIL-18 levels, the AUC-ROC was 0.62 (95%CI: 0.532–0.718) with a cut off point of >900.7 pg/mL having a sensitivity of 51.79% and specificity of 78.57%. This test had a PPV of 70.73% and NPV of 61.97% (Table .2).

Significant factors from the above calculations, cut off points of serum IL-18 (>132 pg/mL) and urine IL- 18 (>900.7 pg/mL) were included in the multiple logistic regression to assess the relationship of these factors to the incidence of AKI in preterm infants, showing that the OR*_adj_* of sIL-18 was 5.89 (95% CI:2.31–15.02), 4.15 (95%CI:1.58–10.89) for uIL-18 (Table 3).

### 4.3. Association of IL-18 promoter polymorphisms with s/uIL-18 levels

The results of the association analysis of −137G > C, −607C > A and −656G > T polymorphisms with sIL-18 and uIL-18 levels in preterm infants showed that only −137G > C and −656G > T polymorphisms significantly affected the level of sIL-18 in preterm infants with AKI (Table 4).

Post-hoc analysis of −137G > C and −656G > T showed that the change of homozygote wild type (G/G) to heterozygote (G/C) of −137G > C polymorphism significantly affected the sIL-18 level (Table 5), with the median sIL-18 level higher in infants with the mutant heterozygous (G/C) than those homozygous wild type (G/G) (166.8 vs 138.4 pg/mL; Table 4). Also, the change of homozygote wild type (G/G) to homozygote mutant (T/T) of −656G > T (rs1946519) significantly affected the sIL-18 level, with a median sIL-18 level higher in infants with homozygous mutant (T/T) than the homozygous wild type (G/G) (198.4 vs 178.8 pg/mL; Table 4).

### 4.4. Association of genotype and allele of IL-18 promoter polymorphisms with AKI in preterm babies

The association analysis of the genotype −137G > C, −607A > C and −656G > T polymorphisms with preterm AKI presented in Table 6 showing that none of the IL-18 promoter polymorphism genotypes was associated with AKI in preterm infants (p > 0.05, OR<1.00). Likewise, there was no association of any of the mutant alleles of the IL-8 promoter polymorphisms with AKI in preterm infants (Table 7). Although it was not significant, there was a trend that the mutant allele slightly increased the risk of AKI in preterm babies as all of the OR values were >1.00 (Table 6).

## 5. Discussion

Inflammation is the most common cause of preterm birth [14–16]. It has been established that IL-18 is involved in the pathogenesis of inflammation, tumours, hemophagocytic syndrome, sepsis, AKI, various autoimmune diseases and cardiac ischemia [28–34]. Serum creatinine is not an ideal biomarker for AKI in preterm infants as it is influenced by maternal creatinine level in the first 48–72 h of life [1–10]. Hence, this study sought to determine the modality of sIL18 and uIL-18 as early biomarkers for AKI in preterm infants and analyse the association of the IL-18 promoter polymorphisms with s-/uIL-18 and the risk of AKI in preterm infants.

In this study, the gestational age and birth weight did not affect the incidence of AKI in preterm infants, which was different from the study of Shalaby et al. (2018) [35] in Saudi Arabia, who showed that gestational age and birth weight affected the incidence of AKI in newborns. This difference may be attributed to our homogenous sample, with the same criteria for subjects with and without preterm AKI, whereas Shalaby et al. included newborns from term until a gestational age of less than 30 weeks [35]. Nonetheless, the level of sIL-18 and uIL- 18 in preterm infants with AKI was significantly higher than that without AKI. Furthermore, multiple logistic regression analysis showed that the cut off of sIL-18 > 132 pg/mL and uIL-18 > 900.7 pg/mL increased the risk of AKI to 5.89 and 4.15 fold, respectively, in preterm infants with AKI compared to those without AKI. The AUC-ROC analysis showed that sIL-18 and uIL-18 could be used as moderate biomarkers to predict AKI in preterm infants, in line with Parikh et al.(2006) [36] and Wang et al. (2017) [37], who reported that IL-18 level can be used as an early biomarker and prognosis for AKI [31,32]. Others study reported similar result with this study, that IL-18 could be used as biomarker for AKI [38–40].

The association analysis of polymorphisms with IL-18 expression showed that infants with −137G > C and −656G > T polymorphisms had significantly higher sIL-18 compared to those with the homozygous wild type. These results show that there is an overlap in the polymorphisms which affect IL-18 expression in AKI preterm infants with those in adult patients with kidney transplantation (−137G > C and −607C > A) and with type 1 diabetes, rheumatoid arthritis and Chron’s disease (−656G > T, −607C > A and −137G > C) [20–28]. A common feature of these phenotypes is the symptoms of chronic inflammation, indicating that these polymorphisms increase the expression of sIL-18 which leads to chronic inflammation, hence is involved in disease development. In this study, both the change from homozygote (G/G) genotype to heterozygote (G/C) of the −137G > C polymorphism and the change of homozygote wild type (G/G) to homozygote mutant (T/T) but not the heterozygous (G/T) of the −656G > T (rs1946519) polymorphism significantly increased sIL-18 expression. However, it is of note that only two infants had the homozygous mutant (C/C) of −137G > C, whereas nineteen infants had the homozygous mutant (T/T) of −656G > T. Nonetheless, the trend of increasing sIL-18 expression from homozygous wild type, heterozygous to homozygous mutants was evident in all polymorphisms, indicating that there is a “dosage-effect of mutant allele”, in which the effect of homozygous mutant to increase the sIL-18 expression is higher than that of heterozygous mutant. Further studies employing a larger sample size are required to confirm this observation.

This study shows that both the genotype and alleles of −137G > C, −607A > C, and −656G > T polymorphisms were not associated with the incidence of AKI in preterm infants. Nonetheless, the analysis of the association of the mutant alleles of the three polymorphisms with the incidence of AKI in preterm infants demonstrated a tendency for mutant alleles to increase the risk of developing AKI as all OR values were above 1.00 (1.34–1.55). It can be assumed that the genetic effect of those polymorphisms on the risk of AKI in preterm infants is weak, hence, the association analysis of 112 samples of both cases and controls is not sufficiently powered to detect a genetic association. In this study, it has been shown that the *IL-18* gene promoter polymorphism is associated with sIL-18, however, AKI is a multifactorial disease influenced by the interactions between multiple susceptibility genes, numerous pro-inflammatory cytokines and environmental factors [37–41]. Most likely, there will not be a single gene or environmental factor that has a large effect on AKI susceptibility. Other risk factors, such as inflammation with increased levels of CRP and immaturity of the kidney itself in preterm infants have a significant role in the occurrence of AKI [42]. Thus, it is not surprising that the effect of *IL-18* gene promoter polymorphisms on the occurrence of AKI is small as its effect on the aetiology of the disease is indirect by regulating the expression of IL-18.

## 6. Conclusion

This study demonstrated that increasing sIL-18 and uIL-18 are risk factors, as well as moderate biomarkers for AKI in preterm infants. The −137G > C and −656G > T polymorphisms affect sIL-18 but not uIL-18, however, none of those polymorphisms are a risk factor for AKI in preterm infants in this population. Larger-scale studies should be conducted to confirm the relationship between IL-18 polymorphisms and the risk of AKI in preterm newborns in Indonesia.

## Supplementary

**Table S1:** Primers for genotyping IL-18 polymorphisms.

No	SNP Target	Primer sequence 5′ → 3′	PCR product size (bp)	TM (°C)
1	−137G > C (rs187238)	Fw: TGACACCATATTGAGCTTGG	562	57.12
		Rv: CAATTCCTTGCTGACTGTCC		58.29
2	−607A > C (rs946518)	Fw: TTTACACTCTGCTCTTCAAACG	572	57.39
3	−656G > T (rs1946519)	Rv: CTTCCTGGTCACACTTCAGC		58.44

Fw: Forward, Rv: Reverse.

**Table S2:** Touch-down PCR programme used for genotyping IL-18 polymorphisms.

No.	Temperature (°C)	Time (minutes)	Cycle
1	94	5	

2	94	1	10 cycles
3	68 reduce 1° **C/cycle**	1	
4	72	1	

5	94	1	25 cycle
6	58	1	
7	72	1	
8	72	5	

9	4	~	

## Figures and Tables

**Table 1:** Characteristics of preterm infants with AKI and without AKI.

Characteristics	AKI		*p* a

	(+) (n =56)	(−) (n =56)	
**Gender**			0.85
Boy	27 (48.2)	26 (44.8)	
Girl	29 (51.8)	27 (47.4)	
**Gestational ages (weeks)**			0.82
Mean (SD)	32.3 (1.4)	32.3 (1.4)	
Median	32	32	
Range	30–36	30–36	
**Birth weight (gr)**			0.970
Mean (SD)	1665.8 (309.4)	1639 (324.7)	
Median	1630	1645	
Range	1000–2210	1100–2320	
**Serum IL-18 (pg/mL)**			<0.001
Mean (SD)	266,8 (366,5)	130,3 (268,9)	
Median	167,4	117,8	
Range	30,7–2118,9	26,5–877,6	
**Urine IL-18 (pg/mL)**			0.010
Mean (SD)	1181,0 (1278,0)	726,9 (600,1)	
Median	918,7	649,8	
Range	156,5–7003,3	96,8–3266,2	

a Gestational age and serum/urine IL-18 Level by Mann–Whitney test; birth weight with t-test; gender using the Chi-square test.

**Table 2:** Diagnostic analysis of serum and urine IL-18 levels as predictor of AKI in preterm infant.

Cut off point	AKI		p^a^	Note

	+	−		
IL-18 serum:			<0.001	Sensitivity 80.36%; Specificity 60.71%; PPV =58.44%; and NPV =75.56%
>132 pg/mL	45	22		
≤132 pg/mL	11	34		
IL-18 urine:			0.001	Sensitivity 51.79%; Specificity 78.57%; PPV =70.73%; and NPV =61.97%
- >900.7 pg/mL	29	12		
- ≤ 900.7 pg/mL	27	44		

a Based on Chi square test.

**Table 3:** The factors associated with the incidence of AKI based on multiple logistic regression analysis.

Variable	Coefficient B	SE (B)	P-value	OR_adj_ (CI 95%)
IL-18 serum (>132 pg/mL)	1.774	0.477	<0.001	5.89 (2.31–15.02)
IL-18 urine (>900,7 pg/mL)	1.423	0.492	0.004	4.15 (1.58–10.89)

Accuracy =85%; R^2^ (Nagelkerke) =39%; OR_adj_ (CI 95%): Odds ratio *adjusted* and Confidence interval 95%.

**Table 4:** Association of IL-18 promoter genotype polymorphisms with sIL-18 and uIL-18.

Polymorphisms	N	%	IL-18 Serum (pg/ml)		*p*	IL-18 Urine (pg/ml)		*p* a
	
			Median	Range		Median	Range	
−137G > C(rs187238)					0.02			0.79
GG	77	67.8	138.4	26.5–2,118		783.5	113.8 –7,003.3	
GC	33	29.6	166.8	58.8–781.8		689.7	96.8–7,003.2	
CC	2	2.6	183.4	173.6–193.3		978.3	241.1–1,292.9	
−607C > A(rs1946518)					0.09			0.70
CC	47	41.74	141	33.2–2,118.9		796.3	113.8–3,266.2	
CA	48	42.61	139.2	26.5–781.8		702	96.8–7,003.2	
AA	17	15.65	166.2	58.8–877.6		711.7	241.1–7,003.3	
−656G > T(rs1946519)					0.05			0.52
GG	47	41.74	198.4	33.2–2,012.7		666	113.8–3,266.2	
GT	46	40.87	141.7	26.5–781.8		702	96.8–7,003.2	
TT	19	17.39	178.8	58.8–2,118.9		666	241.1–7,003.3	

a Kruskal–Wallis test.

**Table 5:** Post-hoc analysis of polymorphism genotype association with serum IL-18.

Polymorphisms	*P* a
−137G > C (rs187238)	
G/G vs G/C	0.01
G/G vs C/C	0.22
G > C vs C/C	0.89
−656G > T (rs1946519)	
G/G vs G/T	0.26
G/G vs T/T	0.02
G/T vs T/T	0.12

a Mann–Whitney test.

**Table 6:** Association of IL-18 polymorphism genotype with AKI among preterm neonates.

Polymorphisms	Preterm Neonates		*P* a	OR (CI 95%)

	AKI (+)	AKI (−)		
	
	(n =56)	(n =56)		
−**137G** > **C (rs187238)**			0.35	
G/G	35	42		0.833 (0.05–13.81)
G/C	20	13		0.538 (0.08–26.82)
C/C	1	1		1.0
−**607C** > **A (rs 946518)**			0.37	
C/C	21	26		0.44 (0.14–1.39)
C/A	24	24		0.55 (0.17–1.71)
A/A	11	6		1.0
−**656G** > **T(rs1946519)**			0.47	
G/G	21	26		0.47 (0.16–1.41)
T/G	23	23		0.58 (0.19–1.75)
T/T	12	7		1.0

a Chi–Square test.

**Table 7:** Association of IL-18 polymorphism alleles with preterm AKI.

Polymorphism alleles	AKI		*P* a	OR (CI 95%)

	(+)	(−)		
	
	(n allele =98) N(%)	(n allele =132) N(%)		
−**137G** > **C (rs187238)**			0.208	1.43 (1.02–2.85)
Allele C	22 (20)	15 (15.2)		
Allele G	90 (80)	97 (84.8)		
−**607C** > **A (rs 946518)**			0.165	1.55 (1.09–2.67)
Allele A	46 (42.8)	36 (32.1)		
Allele C	66 (57.2)	76 (67.9)		
−**656G** > **T (rs1946519)**			0.168	1.34 (1.18–2.30)
Allele T	47(41.8)	37 (33.0)		
Allele G	65 (58.2)	75 (67.0)		

a Chi–Square test.

## References

[1] Stritzke A Thomas S Amin H Fusch C Abhay L Renal consequences of preterm birth Mol Cel Pediatr 2017 4 2 1 9 10.1186/s40348-016-0068-0 PMC5243236 28101838

[2] Viswanathan S Mhanna MJ Acute kidney injury in pre-mature infants J Clin Pediatr 2013 1

[3] Luyckx VA Preterm birth and its impact on renal health Semin Nephrol 2017 37 4 311 9 28711069 10.1016/j.semnephrol.2017.05.002

[4] Bruel A Rozé J-C Quere M-P Flamant C Boivin M Roussey-Kesler G Renal outcome in children born preterm with neonatal acute renal failure: IRENEO—a prospective controlled study Pediatr Nephrol 2016 31 12 2365 73 27335060 10.1007/s00467-016-3444-z

[5] Black MJ Preterm birth and/or factors that lead to preterm delivery: effects on the neonatal kidney J Neonatal Biol 2015 41 1 12

[6] Charlton JR Boohaker L Askenazi L Brophy PD Gien J Incidence and risk factors of early onset neonatal AKI CSAJN 2019 14 2 184 95 10.2215/CJN.03670318 PMC6390916 31738181

[7] Stojanovic V Barisie N Milanovic B Doronjski A Acute kidney injury in preterm infants admitted to a neonatal intensive care unit Pediatr Nephrol 2014 29 11 2213 20 24839217 10.1007/s00467-014-2837-0

[8] Makris K Spanou L Acute kidney injury: definition, pathophysiology and clinical phenotypes Clin Biochem 2016 32 2 85 98 PMC5198510 28303073

[9] Ottonello G Dessì A Neroni P Trudu ME Manus D Fanos V Acute kidney injury in neonatal age J Pediatr Neonatal Individ Med 2014 3 2 2 5

[10] Awdishu L Sheryl E Mohorn PL Huang WT Acute kidney injury CCSAP 2017 BOOK 2.Renal/Pulmonary Critical Care

[11] Durkan AM Alexander RT Acute kidney injury post neonatal asphyxia J Pediatr 2011 158 2 SUPPL e29 33 21238703 10.1016/j.jpeds.2010.11.010

[12] Al Saqladi A-WM Acute kidney Injury : new definitions and beyond J Nephrol Therapeut 2016 61 1 4

[13] Kidney KDIGO Disease improving global Outcomes (KDIGO) clinical practice guideline for acute kidney injury J Int Soc Nephrol 2012 21

[14] Boyle AK Rinaldi SF Norman JE Stock SJ Preterm birth: inflammation, fetal injury and treatment strategies J Reprod Immunol 2017 119 62 6 28122664 10.1016/j.jri.2016.11.008

[15] Koucky M Germanova A Hajek Z Parizek A Kalousova M Kopecky P Pathophysiology of preterm labour Prague Med Rep 2009 110 1 13 24 19591374

[16] Bastek JA Gomez LM Elovitz MA The Role of Inflammation anad Infection in preterm birth Clin Perinatol 2011 38 385 406 21890015 10.1016/j.clp.2011.06.003

[17] Krajewski P Sieroszewski P Karowicz-Bilinska A Kmiecik M Chudzik A Strzalko-Gloskowska B Assessment of interleukin-6, interleukin-8 and interleukin-18 count in the serum of IUGR newborns J Matern Neonatal Med 2014 27 11 1142 5 10.3109/14767058.2013.851186 24093539

[18] Oncel MY Canpolat FE Arayici S Alyamac Dizdar E Uras N Oguz SS Urinary markers of acute kidney injury in newborns with perinatal asphyxia^*^ Ren Fail 2016 38 6 882 8 27055689 10.3109/0886022X.2016.1165070

[19] Li Y Fu C Zhou X Xiao Z Zhu X Jin M Urine interleukin-18 and cystatin-C as biomarkers of acute kidney injury in critically ill neonates Pediatr Nephrol 2012 27 5 851 60 22228436 10.1007/s00467-011-2072-x PMC3315640

[20] Davran F Yilmaz VT Erdem BK Gultekin M Suleymanlar G Akbas H Association of interleukin 18- 607A/C and -137C/G polymorphisms with oxidative stress in renal transplant recipients Ren Fail 2016 38 717 22 26983036 10.3109/0886022X.2016.1158034

[21] Tavares NAC Santos MMS Moura R Araújo J Guimãraes R Crovella S Interleukin 18 (IL18) gene promoter polymorphisms are associated with type 1 diabetes mellitus in Brazilian patients Cytokine 2013 62 2 286 9 23557801 10.1016/j.cyto.2013.03.004

[22] Pawlik A Kurzawski M Drozdzik M Dziedziejko V Safranow K Herczynska M Interleukin18 gene IL18 promoter polymorphisms in patients with rheumatoid arthritis Scand J Rheumatol 2009 38 3 159 65 19229765 10.1080/03009740802600748

[23] Gao SJ Zhang L Lu W Wang L Chen L Zhu Z IL-18 genetic polimorphisms controbute differentially to the susceptibility to Crohn’s diseasae WJG 2015 21 28 8711 22 26229413 10.3748/wjg.v21.i28.8711 PMC4515852

[24] Chen H Zheng W Association of cytokine gene polymorphism with bronchopulmonary dysplacia in Han Chinese Newborn Pediatr Pulmonol 2017 1 7 10.1002/ppul.23902 29136357

[25] Krueger M Heinzmann A Mailaparambil B Härtel C Gäpel W Polymorphisms of interleukin 18 in the genetics of preterm birth and bronchopulmonary dysplasia Arch Dis Child Fetal Neonatal Ed 2011 96 4 299 301 10.1136/adc.2009.174862 20971720

[26] Sadeghi-Bojd S Kordi-Tamandani DM Hashemi M Effect of pro-inflammatory cytokine (IFN-*γ* +874, IL-18-137 G/C,- 607 C/A) genes in relation to risk of vesico-ureteral reflux Ren Fail 2014 361 1 4 10.3109/0886022X.2013.789959 24168677

[27] Sripichai O Fucharoen S Genetic polymorphisms and implications for human diseases special article genetic polymorphisms and implications for human diseases J Med Assoc Thail 2007 2 394 8 17375650

[28] Thompson SR Humphries SE Interleukin-18 genetics and inflammatory disease susceptibility Gene Immun 2007 8 2 91 9 10.1038/sj.gene.6364366 17215860

[29] Dinarello CA Novick D Kim S Kaplanski G Interleukin-18 and IL-18 binding protein Front Immunol 2013 4 10.3389/fimmu.2013.00289 PMC3792554 24115947

[30] Shi B Ni Z Cao L Zhou M Mou S Wang Q Serum IL-18 is closely assosiated with renal tubulointertitial injury and predicts renal prognosis is in IgA neuropathy Hindawi Publishing Corporation 2012 Mediators of Inflamation 2012 ArticleID.728417 10.1155/2012/728417 PMC3306983 22518072

[31] Yamanishi K Mukai K Hashimoto T Ikubo K Nakasho K El-Darawish Y Physiological and Molecular effect of Interleukin-18 administration on the mouse kidney J Transl Med 2018 16 51 29514661 10.1186/s12967-018-1426-6 PMC5842592

[32] Gauer S Sichler O Obermüller N Holzmann Y Kiss E Sobkowiak E IL-18 is expressed in the intercalated cell of human kidney Kidney Int 2007 72 9 1081 7 17687255 10.1038/sj.ki.5002473

[33] Lin X Yuan J Zhao Y Zha Y Urine interleukin-18 in prediction of acute kidney injury: a systemic review and meta-analysis J Nephrol 2014 281 7 16 10.1007/s40620-014-0113-9 PMC4322238 24899123

[34] Dinarello CA Interleukin-18 and the pathogenesis of inflammatory diseases Semin Nephrol 2007 271 98 114 10.1016/j.semnephrol.2006.09.013 17336692

[35] Shalaby M Incidence, risk factors, and outcome of neonatal acute kidney injury: a prospective cohort study Pediatr Nephrol 2018 33 Pt 2 1 8 29869723 10.1007/s00467-018-3966-7

[36] Parikh CR Abraham E Ancukiewicz M Edelstein CL Urine IL-18 is an early diagnostic marker for acute kidney injury and predicts mortality in the intensive care unit J Am Soc Nephrol 2005 16 10 3046 52 16148039 10.1681/ASN.2005030236

[37] Wang Y Bellomo R Cardiac surgery-associated acute kidney injury: risk factors, pathophysiology and treatment Epub 2017 13 11 697 711 10.1038/nrneph.2017.119 28869251

[38] Chan JCM Williams DM Roth KS Kidney failure in infants and children Pediatr Rev 2002 23 2 47 60 11826257 10.1542/pir.23-2-47

[39] Andreoli SP Acute kidney injury in children 2009 253 63 10.1007/s00467-008-1074-9 PMC2756346 19083019

[40] Selewski DT Charlton JR Jetton JG Guillet R Neon Acute Kidn Inj 2015 136 2 10.1542/peds.2014-3819 26169430

[41] Jetton JG Askenazi DJ Update on acute kidney injury in the neonate Curr Opin Pediatr 2012 24 2 191 6 22227783 10.1097/MOP.0b013e32834f62d5 PMC5545784

[42] Tang Y Kwong-Mak S Xu AP Yao Lan H Role of c-reactive protein in the pathogenesis of acute kidney injury Nephrology 2008 50 52 10.1111/nep.13454 30298655

